# Lessons in Protein Design from Combined Evolution and Conformational Dynamics

**DOI:** 10.1038/srep14259

**Published:** 2015-09-21

**Authors:** Swarnendu Tripathi, M. Neal Waxham, Margaret S. Cheung, Yin Liu

**Affiliations:** 1 Department of Physics, University of Houston, Houston, TX; 2 Department of Neurobiology and Anatomy, University of Texas, Health Science Center, Houston, TX; 3 Center for Theoretical Biological Physics, Rice University, Houston, TX.

## Abstract

Protein-protein interactions play important roles in the control of every cellular process. How natural selection has optimized protein design to produce molecules capable of binding to many partner proteins is a fascinating problem but not well understood. Here, we performed a combinatorial analysis of protein sequence evolution and conformational dynamics to study how calmodulin (CaM), which plays essential roles in calcium signaling pathways, has adapted to bind to a large number of partner proteins. We discovered that amino acid residues in CaM can be partitioned into unique classes according to their degree of evolutionary conservation and local stability. Holistically, categorization of CaM residues into these classes reveals enriched physico-chemical interactions required for binding to diverse targets, balanced against the need to maintain the folding and structural modularity of CaM to achieve its overall function. The sequence-structure-function relationship of CaM provides a concrete example of the general principle of protein design. We have demonstrated the synergy between the fields of molecular evolution and protein biophysics and created a generalizable framework broadly applicable to the study of protein-protein interactions.

Optimized protein design for multiple protein binding partners (promiscuity) requires a balance between structural stability and flexibility^1^. Structural stability provides architectural framework while flexibility provides for adaptable surfaces or enzymatic sites to mediate function^2^. Both aspects of protein design must have a degree of adaptability to adjust to pressures from evolutionary advances^3^,^4^. However, it remains elusive how a protein evolves under the selection constraint for versatility over stability in order to achieve a functionally optimized structure. This is a fundamental question for understanding the impact of evolutionary pressure on a protein sequence and how resulting mutations are tolerated or not in the face of meeting demands of conformational dynamics required for function^5^,^6^,^7^. Recent advances in understanding protein folding and dynamics employing energy landscape theory^8^,^9^ have provided a framework to quantify this subtle complexity. For efficient protein folding, evolutionary pressure selects for amino acids that provide a unique folded state on the energy landscape^10^ or a smooth funnel-like energy landscape^11^, so the folding pathway avoids long-lived kinetic traps^12^ (termed “minimally frustrated”). In contrast, the least energetically favorable residues (termed “highly frustrated”) are typically related to the functional sites of proteins, so the frustration could be a functionally useful adaptation and contribute to the binding and allosteric properties of a protein^13^. Accordingly, the knowledge from both protein evolution and energy landscape theory provides synergistic potential to unravel the underlying mechanisms dictating the structure and function of proteins. In this study, we performed a combinatory analysis of protein sequence evolution and local energetic frustration to identify how calmodulin (CaM) has balanced diversification during evolution.

CaM is a remarkable example of a multi-specific binding protein that plays crucial roles in intracellular calcium (Ca^2+^) signaling by regulating a wide array of downstream partner proteins^14^. The sequence of CaM comprises 148 amino acids, more than 60% of which are conserved among eukaryotes and 100% conserved among vertebrates^15^. While relatively small, CaM is densely packed with functional sites. There are four EF-hand (helix-loop-helix) motifs for Ca^2+^ binding separated into two lobes that are connected by a flexible tether^16^. A distinct binding pocket on each of the lobes accommodates target protein binding and CaM’s promiscuity for interacting with hundreds of different targets depends on its remarkable structural plasticity^17^. Upon binding to Ca^2+^, CaM interacts with numerous protein kinases, including CaM-dependent protein kinase I, II, and IV, phosphorylase kinase, myosin light chain kinase, and the protein phosphatase calcineurin. It also regulates cell-signaling proteins, such as nitric oxide synthases and cyclic nucleotide phosphodiesterase. In addition, it interacts with cytoskeletal proteins to modulate cell movement and growth. CaM can also bind in its apo-form (Ca^2+^-free) to some targets, such as the neuronal proteins neuromodulin and neurogranin^18^. As a consequence of CaM’s importance in regulating cellular function, an immense amount of structural information has emerged that provide a unique opportunity for analysis^19^. Because CaM is optimized through evolution to bind to a multitude of diverse targets^4^,^20^, we first determined the evolutionary conservation of its amino acid residues using the Evolutionary Tracer^21^. We then quantified the conformational dynamics in terms of local frustration of amino acids in CaM in 60 CaM/target complexes using the Frustratometer^22^. With this unique combinatorial approach, we were able to separate CaM residues into novel discrete classes, bringing significant new insights of how evolution has optimized CaM to balance promiscuous binding behavior, while maintaining specificity.

## Results

### Combinatorial analysis of evolution and energetic frustration can classify CaM residues into unique categories

**Figure 1:**
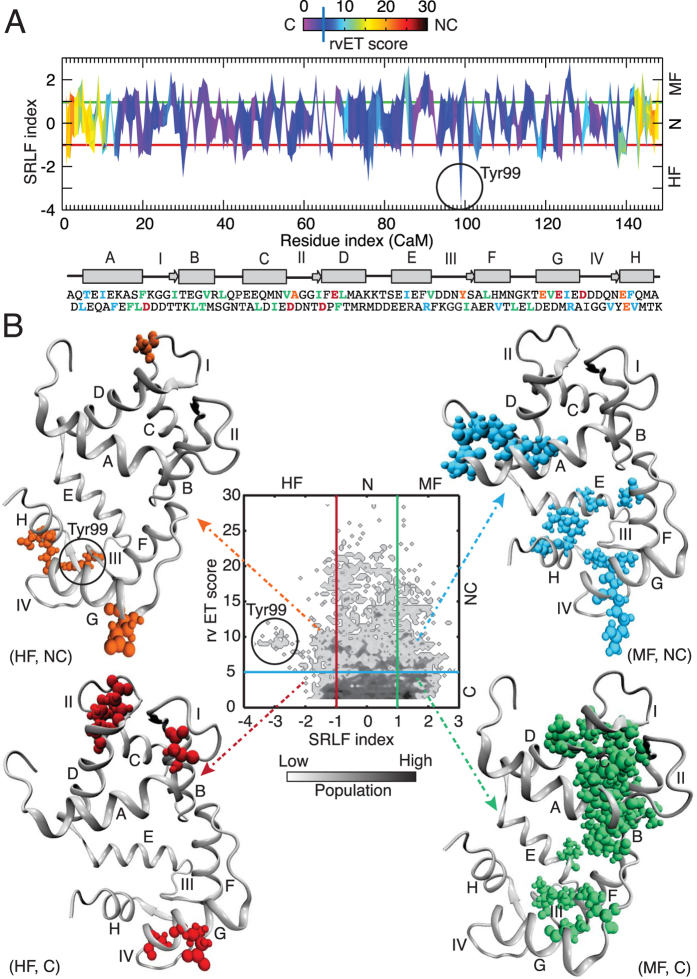
Distributions of evolutionary trace and local frustration of the CaM residues in the target-bound complexes. (**A**) Two-dimensional map plot of real-value evolutionary trace (rvET) score vs. single residue level frustration (SRLF) index along the CaM residues. (**B**) Two-dimensional histogram plot of rvET score vs. SRLF index of the CaM residues. Based on the evolutionary analysis, CaM residues are divided as conserved (C, rvET score < 5) and non-conserved (NC, rvET score > 5). Based on the local frustration analysis, CaM residues are divided as highly frustrated (HF, SRLF index <−1), minimally frustrated (MF, SRLF index > 1) and neutral (N, −1 < SRLF index < 1). From the combinatorial analysis of evolution and frustration, CaM residues are divided in six groups: (MF, C); (HF, C); (HF, NC); (MF, NC); (N, C); and (N, NC) in (**B**). The secondary structure of CaM is shown below Panel (**A**) with the sequence in one letter amino-acid code. In the secondary structure of CaM, 8 helices are shown in rectangle from A to H. The 4 Ca^2+^-binding loops are indicted from I to IV. The CaM residues in the sequence are colored based on the rvET score and SRLF index: red (HF, C), orange (HF, NC), cyan (MF, NC) and green (MF, C) in bold letters. Similarly, the residues that belong to (HF, C), (HF, NC), (MF, NC) and (MF, C) classes are colored accordingly in the three-dimensional structure of CaM (from CaM-CaMKI complex) and represented in spheres. Residue Tyr99 of CaM from (HF, NC) class is indicated by circle.

We began with an evolutionary analysis of more than 300 homologous sequences of CaM that divided the CaM residues into two distinct groups: conserved and non-conserved (Figure S1A and see Methods). To explore how CaM’s sequence has adapted during evolution to diversify its function through conformational dynamics, we then analyzed 60 CaM/target complexes available in the protein data bank (PDB) (Table S1). This quantitative analysis of local frustration revealed that CaM residues could be categorized as minimally frustrated (energetically favorable), highly frustrated (energetically unfavorable) or neutral (neither favorable nor unfavorable)^23^ (Figure S1B and see Methods). By annotating the energetic frustration indices along the CaM sequence with its evolutionary conservation, one amino acid at a time (Fig. 1A), each residue of CaM could be separated into one of six unique classes: (i) minimally frustrated and conserved (MF, C); (ii) highly frustrated and conserved (HF, C); (iii) highly frustrated and non-conserved (HF, NC); (iv) minimally frustrated and non-conserved (MF, NC); (v) neutral and conserved (N, C); and (vi) neutral and non-conserved (N, NC) (Fig. 1B). We observed that majority of the CaM residues fall into (N, C) and (N, NC) classes composing 32% and 22% of the population, respectively. On the contrary, the CaM residues in the (HF, NC) class represent the lowest population (3.3%). The detailed classification of CaM residues can be found in the Supplementary Table S2.

### Conserved residues make up the protein folding scaffolds

The conserved residues can be minimally frustrated, highly frustrated, or neutral. We found that the minimally frustrated and conserved residues (Fig. 1B) constitute the helices in the four EF-hands of CaM and are all hydrophobic (except one polar residue, T34) (Table S2). These residues offer essential hydrophobic forces that drive folding and maintain stability of the CaM sequence.

All of the highly frustrated and conserved residues are negatively charged and are distributed on the four Ca^2+^ binding loops, which lie between the two helices of an EF hand (Table S2). Specifically, the Asp residues at the first position in each of the four Ca^2+^-binding loops of CaM, which coordinate with the Ca^2+^ ion, belong to the (HF, C) group (see Figure S2). Typically, frustrated residues are found at the sites of protein-protein interaction and/or catalytic sites and can also be described as areas of high conformational flexibility^24^,^25^. Therefore, these energetically frustrated amino acids located at the loops are essential to facilitate the binding and release of Ca^2+^. While they are evolutionarily conserved, their high frustration (conformational flexibility) at the residue level reflects the structural dynamics required for accommodating Ca^2+^-binding. These conserved EF hands and their Ca^2+^-binding loops form a structurally common scaffold of CaM along the evolutionary history. We note that the frustratometer algorithm excludes all the heteroatoms from protein or protein complexes^22^ and as a result, Ca^2+^ ions were not involved in the frustration analysis of the CaM complexes. Hence, a future investigation is required to understand the correlation between local frustration indices in the Ca^2+^-binding loops and their effect on Ca^2+^-binding affinity.

### Highly frustrated and non-conserved residues contribute to expanded protein functions in higher eukaryotes

The highly frustrated and non-conserved class of residues in CaM composes 3.3% of the amino acids. Despite their low numbers, their roles in the evolutionary development of specific functions of CaM appear essential. Specifically, a unique tyrosine residue at position 99 (Y99) was immediately identified because it is the most frustrated residue in CaM and non-conserved (shown by a circle in Fig. 1A,B). When Y99 was assessed from an evolutionary perspective, we also discovered that this residue exhibits diversity; amino acid 99 is Leu in CaM from baker’s yeast and Phe in CaM from fruit fly or barley (Figure S3). These mutations lead to a significant decrease in its frustration level (Figure S5) in lower eukaryotes. Interestingly, Y99 rarely forms direct contacts with the targets; it is located at the opposite side of the CaM-target interface making it available as a substrate for protein tyrosine kinase phosphorylation^26^. Modifications at this position induce a conformational change that leads to altered CaM binding^27^.

In addition, we found the negatively charged amino acids (E139 and E140) in the fourth Ca^2+^ binding loop are highly frustrated and non-conserved relative to all other negatively charged residues in CaM (Table S2). In particular, residue E140 at the twelfth position of the Ca^2+^-binding loop IV coordinates with Ca^2+^ ion (Figure S2). It was noted that when E139 and E140 are replaced by Gln in baker’s yeast, it resulted in the loss of Ca^2+^-binding to this site of CaM. Therefore, the evolutionary pressure to select for Glu at residues 139 and 140 correlates with a gain of function in Ca^2+^-binding to EF-hand IV, thus expanding the ability to decode a wider range of Ca^2+^ signals in higher eukaryotes.

### Minimally frustrated and non-conserved amino acids make up modular scaffolds

This class of residues contains nine hydrophobic residues, one polar residue, and two positively charged residues (Table S2). These minimally frustrated residues are typically located at the CaM-target binding interface (Fig. 1B) and thus provide structural modularity in the overall design. These non-conserved residues appear to enrich the local physico-chemical properties to fine-tune the specificity of target binding over the evolutionary process without altering the local protein-folding scaffold. For example, we found the positively charged residue R126 is among the least frustrated residues in all the complexes, but when it is replaced by Lys, as in soybean CaM, its frustration level increased significantly (Figure S6A). The correlation between the residue replacement and frustration variation was also observed for residues R90, which is minimally frustrated in 53 out of 58 complexes examined but become neutral when replaced by K90 in yeast or soybean (Figure S6B).

### Structural and functional importance of the neutral residues

An earlier study by Ferreiro *et al.*^23^ demonstrated that irrespective of the protein size nearly 50% of amino-acid residues are neutral (based on the single residue level frustration) and randomly distributed in the structure. In particular, Thr, Ser and Gly residues are almost always found to be neutral in frustration analyses^23^. This phenomenon is also observed in CaM, where the majority of Thr and Ser residues (14 out of 16) are neutral (Table S2). Among them, Thr28, Thr29, Ser38 and Ser101 are evolutionarily conserved, while the remainders are non-conserved. Additionally, majority of the Gly residues (7 out of 11) of CaM belong to the (N, C) class (Table S2). These Gly residues are mostly located at the Ca^2+^-binding loops and the linkers connecting the EF-hands of CaM, and exhibit high flexibility in the target-unbound apo- and holo-states of CaM^28^.

### The Methionine residues support target-binding promiscuity and comprise the functional scaffolds

**Figure 2:**
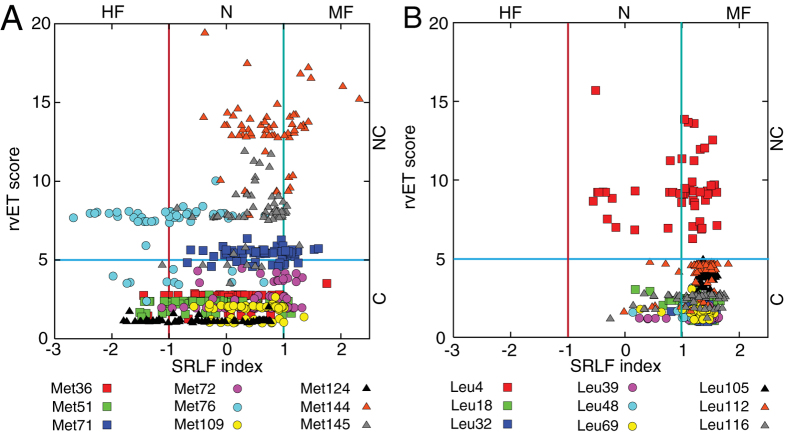
Evolutionary trace and local frustration of Met and Leu residues of CaM. (**A**) rvET score vs. SRLF index plot of nine Met residues of CaM at positions 36, 51, 71, 72, 76, 109, 124 144, and 145. (**B**) rvET score vs. SRLF index plot of nine Leu residues of CaM at positions 4, 18, 32, 39, 48, 69, 105, 112, and 116.

There are four symmetrically positioned Met residues in each domain of CaM and one resides in the central linker in vertebrates. Based on the combinatorial analysis results, we discovered that the Met residues of CaM span the entire range of frustration levels among the different CaM-target complexes. The frustration analysis revealed that Met residues at positions of the N-domain (36, 71, 72) and the C-domain (109, 144 and 145) are mostly neutral or minimally frustrated and likely provide a set of core essential binding residues for target interaction. In contrast, M51 (at the N-domain), M76 (at the linker), and M124 (at the C-domain) are mostly neutral, but can be highly frustrated (Fig. 2A) in a subset of complexes. Not only do the frustration levels of the Met residues vary widely, but they also demonstrate different degrees of conservation. The evolutionary analysis showed that M36, M51, M72, M109, and M124 are among the conserved residues in CaM, while other Met residues are not conserved (Fig. 2A).

We demonstrated that these nine Met residues indeed behave differently in both conformational dynamics and evolution than other hydrophobic residues. By performing the same combinatorial analysis on the nine Leu residues in CaM, we found they are either minimally frustrated or neutral (Fig. 2B) and are more conserved (except L4) than Met residues. Therefore, it is not surprising to find when Met residues were replaced by the hydrophobic residues without a thioester group in some lower eukaryotes, their frustration levels decreased. This can be seen when Met was replaced by Ile at position 36 for CaM (as in soybean) (Figure S7A), Leu at positions 51 (as in baker’s yeast) (Figure S7B), Leu at position 71 (as in barley) (Figure S7C), Leu at positions 109 (Figure S8A) and 145 (Figure S8D) (as in baker’s yeast), or Val at position 144 (as in barley) (Figure S8C). These findings suggest that Met residues are selected through evolution to increase binding promiscuity in higher eukaryotes.

To explore the effect of the targets on the calculated frustration index of individual Met residues in CaM, we separated the targets from the CaM-target complexes and re-computed the frustration (Figure S7 and S8) (see Methods). Thus any difference in the frustration levels of Met residues between the presence and the absence of the target can be attributed to the target at the binding interface with CaM. Interestingly, our comparative analysis revealed that the targets are rarely associated with the frustration level of Met, except M51 and M124 residues in a subset of complexes. The frustration level of M51 decreases upon target binding in about 15% of complexes (Figure S7). The profile of M124 was found to be particularly intriguing. While its frustration level significantly decreases in nearly 25% of complexes, for another 7% of complexes its frustration increases upon target binding (Figure S8). This suggests that targets contribute to a wide range of frustration of M124.

**Figure 3:**
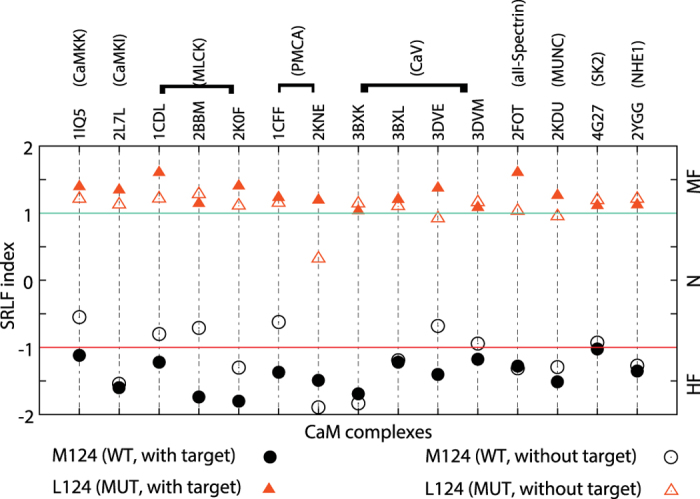
The effect of the targets on the frustration of Met124 in CaM complexes. SRLF index of Met124 in the wild-type (WT) and Leu124 in the mutant (MUT; based on homology modeling) of CaM in the presence and absence (simply by separating the targets from CaM in the bound complexes) of the target are shown. The plot represents 15 CaM complexes for 9 different targets (CaMKK (Ca^2+^/CaM-dependent kinase kinase), CaMKI (Ca^2+^/CaM-dependent kinase I), MLCK (Ca^2+^/CaM-dependent myosin light chain kinase), PMCA (plasma membrane Ca^2+^-ATPase), CaV (voltage-activated Ca^2+^-channel), alphaII-spectrin, MUNC (mammalian uncoordinated proteins), SK2 (small conductance calcium-activated potassium 2 channel), and NHE1 (sodium/hydrogen exchanger 1)) in which Met124 is highly frustrated in the WT CaM for the target-bound complexes.

In addition, we found M124 is one of the most conserved residues in CaM compared to its diverse frustration level. We evaluated how M124 evolved to accommodate a variety of targets by exploring its evolutionary history (Figure S9). Our analysis showed that only two variants, Leu and Ile exist at this position in some lower eukaryotes (e.g. *A. aegypti* and *C.elegans*). Based on homology modeling of CaM complexes where M124 is highly frustrated (see Methods), we showed that when Leu124 replaced Met124, its frustration decreased significantly (more than two-fold change is observed, see Fig. 3). Moreover, the frustration level of Leu residue at this position was not significantly changed between the presence and absence of the target. These findings revealed that Met124 has evolved against a singular notion of stability. Instead, its conformational dynamics is a tradeoff for binding promiscuity to diversify CaM’s function, contributing to the “functional scaffolds” in higher eukaryotes.

## Discussion

Proteins are eminently evolvable due to their ability to adapt in response to mutation and the pressure from selection^29^,^30^. There is a recent trend in integrating evolution with chemical and physical properties of proteins to better investigate protein sequence landscape and functions^31^,^32^. A classical view of proteins according to their biochemical properties of amino acids noted evolutionary conservation of hydrophobic residues in the core of the proteins^33^. The authors later refined the finding that a steady decline in proteome hydrophobicity (oil escape) may explain the emergence of intrinsically disordered proteins, which exhibit conformational diversity^34^. Tokuriki and Tawfik^1^ further pointed out that the promiscuous proteins requires conformational diversity under evolutionary pressure. Sikosek and Chan^6^ then stressed the importance of protein biophysics as one of the possible evolutionary constraints for proteins with marginal stability. However, to the best of our knowledge, there has not previously been an approach that quantifies protein stability at the residue level in the context of evolution to evaluate how the balance between conformational stability and flexibility has been optimized through evolution.

In this study, we developed an approach to integrate protein evolution and dynamics and applied it to a multi-target binding protein, CaM. What fascinates us most about CaM is it is able to bind to hundreds of targets with high specificity, yet the amino acid sequences of CaM share great identity in eukaryotes and are completely invariant among all vertebrates^15^ (also see supplementary information for the sequence alignment in Figure S3). This indicates that CaM in vertebrates has been functionally optimized through evolution and/or that there is no tolerance for additional mutations. Our analyses that incorporate protein biophysics and evolution showed that there were three basic categories of “scaffold” amino acid residues in CaM. For the first group, residues that are minimally frustrated and conserved are typically buried in protein interiors and help form critical secondary structural elements to build the protein-folding scaffold. The second group was termed “modular” because residues in this group are generally located at the CaM-target interface. They are minimally frustrated but not evolutionarily conserved. They have the ability to change local chemistry without affecting the overall folding scaffold. The third group of amino acids was termed the “functional scaffolds”. This class of amino acids contains the Met residues that are known to be important in forming contacts with different target proteins.

It is interesting to note that for proteins with known structures, Met is a relatively rare amino acid accounting for only 1.5% of all the residues or 4.4% when only the hydrophobic residues (Ala, Val, Leu, Ile, Phe and Met) are considered^35^. However, CaM is exceptionally rich in Met that represent 6% of all the residues or 18% considering only the hydrophobic residues. In fact 46% of the accessible surface area of the hydrophobic patches of CaM important for target interactions is contributed by Met residues^35^. A wide variety of frustration level in Met residues is paramount for CaM to be able to accommodate binding to its hundreds of different target proteins. Evolutionarily, Met replaces other highly hydrophobic residues in CaM as a tradeoff of stability (robustness) to gain local flexibility (disorder/adaptivity) in order to interact with additional partners. Beside the Met residues, the Lys residues of CaM also display similar behavior in terms of the variation in their frustration level and degree of conservation (Figure S10). Lys accounts for about 5.4% of all residues in the CaM sequence, but unlike the extraordinarily rich Met in CaM, the frequency of Lys residues is generally high in protein sequences, accounting for about 6.7% of all residues in proteins with known structures^36^. For this reason, the specific role of Lys residues in target binding is an interesting future research direction.

Holistically, within the context of these three basic scaffolds, amino acid mutations were selected and tolerated through evolution (from lower eukaryotes to vertebrates) to balance the need for accommodating additional binding targets within the structural/functional framework of the protein. However, this mechanism of achieving binding promiscuity through amino acid variation appears to have reached its limit as all vertebrate CaMs share the identical amino acid sequence. Still, CaM has continued to expand its promiscuity as demands on the target selection and binding affinity of CaM have continued to increase. Selective post-translational modifications (PTM) on a few key amino acids appear to fill this role. One such mechanism, highlighted by our analysis, is at amino acid Tyr99. Tyr99 appears in the evolutionary tree at the transition between drosophila and fish and was identified as the most “frustrated” residue in CaM. Remarkably, Tyr99 is a target for tyrosine kinase mediated post-translational modification, and phosphorylation alters CaM’s target binding affinity and specificity^37^. We propose that evolutionary pressure continued to drive the need for CaM to adopt increased dynamics for tuning its target binding capacity and it usurped the tyrosine kinase pathway to overcome the inability to tolerate additional amino acid mutations. Besides Tyr99, it is also known that Met residues undergo PTM. For example, Met124 is susceptible to oxidation and oxidation alters the binding properties of CaM^38^. It appears that vertebrates have utilized alternative mechanisms to tune CaM’s binding properties through oxidation as well as phosphorylation^37^,^38^.

In this study, we quantified the local frustration of amino acids on CaM based on the available structures of CaM complexes from the PDB. These structures do not consider the conformational dynamics involved in target binding. We have previously shown a binding mechanism of CaM with its targets that undergoes a process of “conformational and mutually induced fit”^39^. Others also stated the importance of structural flexibility in binding^40^,^41^. We speculate that frustration that arises during the binding process between CaM and target^42^ also serves as a possible evolutionary constraint for CaM’s promiscuity and target specificity. Future studies will focus on identifying co-evolved residues from both CaM and targets to infer spatial contacts^43^ and quantifying such frustration in the co-evolved residues^44^.

The framework developed in this study synergistically brings protein evolution and conformational dynamics together and can be generalized to study other promiscuous proteins to understand the underlying mechanisms for how these molecules achieve target selectivity to modulate specific biological functions. Some well-known examples of promiscuous proteins are caveolins^45^, β-arrestins^46^, Ras^47^, TP53^48^ and ubiquitin^49^ that play important roles in specific cellular signaling cascades. Promiscuous proteins are important for the robustness of metabolic and signaling networks in the cell^50^, so understanding the general rules governing their multi-specificity in protein-protein interactions would greatly assist in unraveling the mechanisms that dictate pathway decisions. Our analysis is not dependent on the amino acid composition of the protein; instead, the classification of the amino acids is based on their frustration levels and degrees of conservation. The basic principles on the role of frustration and evolutionary conservation in protein binding will offer clues on how the proteins have been evolved to achieve their binding multi-specificity and diverse biological functions. The amino acid residues identified from the combinatorial analysis can serve as guides for designing novel proteins or small ligands for manipulating protein binding and function.

## Methods

### Structural preparation of calmodulin complexes

We examined the structures of 60 calmodulin/target complexes representing 24 different targets determined by x-crystallography or NMR from the Protein Data Bank (PDB) (Table S1) using the list in ref. 19. We included the structures of CaM with small target peptides, as well as large target proteins and only structures containing full-length CaM. For ensemble structures from NMR either the best representative conformer in the ensemble structure or the structure of the first model was used. We removed the heteroatoms and the hydrogen atoms for residues from the structures of CaM complexes for all of the calculations.

### Evolutionary analysis of calmodulin residues

We analyzed the degree of conservation for each residue of CaM. We followed the procedure by Mihalek *et al.*^21^ using the Evolutionary Trace server (http://mammoth.bcm.tmc.edu/ETserver.html). Protein sequences that are homologous to CaM were obtained with BLAST search against the NCBI Entrez non-redundant protein sequence database, with the Expect (E) value smaller than 0.05. The sequences identical to human CaM, or the short sequences with a length less than 0.8 times the length of human CaM were removed from the sequence set. The multiple sequence alignment (MSA) was performed with ClustalW and then the MSA analysis results were subject to a phylogenetic tree construction. The assumption of the current method is that the more functionally important the residue, the sooner it becomes fixed in the evolutionary tree branches. The real-value Evolutionary Trace (rvET) scores for each residue in CaM were calculated based on the phylogenetic tree and were used to indicate the degree of conservation of the residue. A cutoff of 5 for conservation was selected based on the distribution of the rvET scores of all CaM residues (Figure S1A). So a residue is defined as conserved if its corresponding rvET score is less than 5, otherwise, it is considered as non-conserved.

### Analysis of local frustration in calmodulin from complexes

We used the Fereiro-Wolynes algorithm^23^ for the calculation of local energetic frustration in the CaM complexes. We computed the single residue level frustration (SRLF) index from the ‘frustratometer’ server (http://www.frustratometer.tk). The server estimates the energy of a protein complex and compares it to the energies of a set of ‘decoy’ states. Only the most common 20 amino acids were taken into account. Our analysis of frustration in the CaM complexes was based on the single residue level ‘mutational frustration’. In this criterion, decoys are constructed from the mutations of single residue by randomly selecting the amino acid identities in the native state. The local SRLF index for each amino acid is defined as a *Z*-score of the native state energy in the single residue level compared to the *N* decoys. For the single residue mutational frustration a residue is defined as stabilizing or minimally frustrated if its SRLF index defined by a *Z*-score is >1 (Figure S1B). On the other hand, a residue is defined as destabilizing or highly frustrated if its SRLF index <−1^23^. Otherwise, a residue is defined as neutral if the SRLF index is between the above limits. Note that the interactions of the residues that are in physical contact are also affected by the presence of residues in the binding partner. For the calculation of the SRLF of CaM in the absence of the target, we first removed the structure of the target from CaM complexes and repeated the same procedure as described above to calculate the SRLF for each residue of CaM in the non-target bound state.

### Homology modeling of mutated CaM complexes

We performed homology modeling to construct the mutated CaM-target complex structures of the M124/L124 amino-acid variant (of CaM). HOMCOS (Homology Modeling of Complex Structure) server^51^ was used to obtain the alignment and script files along with the template PDB file for the MODELLER^52^ (version 9.14) program to build a full atomic model of complex structures for the M124/L124 amino-acid variant. The wild-type structure of the CaM complexes was used as the homologous template in MODELLER.

## Additional Information

**How to cite this article**: Tripathi, S. *et al.* Lessons in Protein Design from Combined Evolution and Conformational Dynamics. *Sci. Rep.* **5**, 14259; doi: 10.1038/srep14259 (2015).

## Supplementary Material

Supplementary Information

## References

[b1] Tokuriki N. & Tawfik D. S. Protein dynamism and evolvability. Science 324, 203–207 (2009). 19359577 10.1126/science.1169375

[b2] Boehr D. D., Nussinov R. & Wright P. E. The role of dynamic conformational ensembles in biomolecular recognition. Nat Chem Biol 5, 789–796 (2009). 19841628 10.1038/nchembio.232 PMC2916928

[b3] Ma B., Wolfson H. J. & Nussinov R. Protein functional epitopes: hot spots, dynamics and combinatorial libraries. Curr Opin Struct Biol 11, 364–369 (2001). 11406388 10.1016/s0959-440x(00)00216-5

[b4] Shifman J. M. & Mayo S. L. Exploring the origins of binding specificity through the computational redesign of calmodulin. Proc Natl Acad Sci USA 100, 13274–13279 (2003). 14597710 10.1073/pnas.2234277100 PMC263780

[b5] Skolnick J. & Gao M. Interplay of physics and evolution in the likely origin of protein biochemical function. Proc Natl Acad Sci USA 110, 9344–9349 (2013). 23690621 10.1073/pnas.1300011110 PMC3677488

[b6] Sikosek T. & Chan H. S. Biophysics of protein evolution and evolutionary protein biophysics. J R Soc Interface 11, 20140419 (2014). 25165599 10.1098/rsif.2014.0419 PMC4191086

[b7] Kinch L. N. & Grishin N. V. Evolution of protein structures and functions. Curr Opin Struct Biol 12, 400–408 (2002). 12127461 10.1016/s0959-440x(02)00338-x

[b8] Bryngelson J. D. & Wolynes P. G. Spin glasses and the statistical mechanics of protein folding. Proc Natl Acad Sci USA 84, 7524–7528 (1987). 3478708 10.1073/pnas.84.21.7524 PMC299331

[b9] Dill K. A. Polymer principles and protein folding. Protein Sci 8, 1166–1180 (1999). 10386867 10.1110/ps.8.6.1166 PMC2144345

[b10] Sun S., Brem R., Chan H. S. & Dill K. A. Designing amino acid sequences to fold with good hydrophobic cores. Protein Eng 8, 1205–1213 (1995). 8869633 10.1093/protein/8.12.1205

[b11] Leopold P. E., Montal M. & Onuchic J. N. Protein folding funnels: a kinetic approach to the sequence-structure relationship. Proc Natl Acad Sci USA 89, 8721–8725 (1992). 1528885 10.1073/pnas.89.18.8721 PMC49992

[b12] Kubelka J., Hofrichter J. & Eaton W. A. The protein folding 'speed limit'. Curr Opin Struct Biol 14, 76–88 (2004). 15102453 10.1016/j.sbi.2004.01.013

[b13] Ferreiro D. U., Komives E. A. & Wolynes P. G. Frustration in biomolecules. Quarterly Reviews of Biophysics 47, 285–363 (2014). 25225856 10.1017/S0033583514000092 PMC4256721

[b14] Yap K. L. *et al.* Calmodulin target database. J Struct Funct Genomics 1, 8–14 (2000). 12836676 10.1023/a:1011320027914

[b15] Davis T. N. & Thorner J. Vertebrate and yeast calmodulin, despite significant sequence divergence, are functionally interchangeable. Proc Natl Acad Sci USA 86, 7909–7913 (1989). 2554295 10.1073/pnas.86.20.7909 PMC298181

[b16] Berchtold M. W. & Villalobo A. The many faces of calmodulin in cell proliferation, programmed cell death, autophagy, and cancer. Biochim Biophys Acta 1843, 398–435 (2014). 24188867 10.1016/j.bbamcr.2013.10.021

[b17] Meador W. E., Means A. R. & Quiocho F. A. Modulation of Calmodulin Plasticity in Molecular Recognition on the Basis of X-Ray Structures. Science 262, 1718–1721 (1993). 8259515 10.1126/science.8259515

[b18] Yamniuk A. P. & Vogel H. J. Calmodulin's flexibility allows for promiscuity in its interactions with target proteins and peptides. Mol Biotechnol 27, 33–57 (2004). 15122046 10.1385/MB:27:1:33

[b19] Tidow H. & Nissen P. Structural diversity of calmodulin binding to its target sites. FEBS J 280, 5551–5565 (2013). 23601118 10.1111/febs.12296

[b20] Fromer M. & Shifman J. M. Tradeoff between stability and multispecificity in the design of promiscuous proteins. PLoS Comput Biol 5, e1000627 (2009). 20041208 10.1371/journal.pcbi.1000627 PMC2790338

[b21] Mihalek I., Res I. & Lichtarge O. A family of evolution-entropy hybrid methods for ranking protein residues by importance. J Mol Biol 336, 1265–1282 (2004). 15037084 10.1016/j.jmb.2003.12.078

[b22] Jenik M. *et al.* Protein frustratometer: a tool to localize energetic frustration in protein molecules. Nucleic Acids Res 40, W348–351 (2012). 22645321 10.1093/nar/gks447 PMC3394345

[b23] Ferreiro D. U., Hegler J. A., Komives E. A. & Wolynes P. G. Localizing frustration in native proteins and protein assemblies. Proc Natl Acad Sci USA 104, 19819–19824 (2007). 18077414 10.1073/pnas.0709915104 PMC2148382

[b24] Ferreiro D. U., Hegler J. A., Komives E. A. & Wolynes P. G. On the role of frustration in the energy landscapes of allosteric proteins. Proc Natl Acad Sci USA 108, 3499–3503 (2011). 21273505 10.1073/pnas.1018980108 PMC3048099

[b25] Li W., Wolynes P. G. & Takada S. Frustration, specific sequence dependence, and nonlinearity in large-amplitude fluctuations of allosteric proteins. Proc Natl Acad Sci USA 108, 3504–3509 (2011). 21307307 10.1073/pnas.1018983108 PMC3048140

[b26] Koster S., Pavkov-Keller T., Kuhlbrandt W. & Yildiz O. Structure of human Na+/H+ exchanger NHE1 regulatory region in complex with calmodulin and Ca2+. J Biol Chem 286, 40954–40961 (2011). 21931166 10.1074/jbc.M111.286906 PMC3220496

[b27] Panina S. *et al.* Significance of calcium binding, tyrosine phosphorylation, and lysine trimethylation for the essential function of calmodulin in vertebrate cells analyzed in a novel gene replacement system. J Biol Chem 287, 18173–18181 (2012). 22493455 10.1074/jbc.M112.339382 PMC3365693

[b28] Tripathi S. & Portman J. J. Inherent flexibility determines the transition mechanisms of the EF-hands of calmodulin. Proc Natl Acad Sci USA 106, 2104–2109 (2009). 19190183 10.1073/pnas.0806872106 PMC2650115

[b29] Romero P. A. & Arnold F. H. Exploring protein fitness landscapes by directed evolution. Nat Rev Mol Cell Biol 10, 866–876 (2009). 19935669 10.1038/nrm2805 PMC2997618

[b30] Musso G., Emili A. & Zhang Z. Characterization and evolutionary analysis of protein-protein interaction networks. Methods Mol Biol 856, 363–380 (2012). 22399467 10.1007/978-1-61779-585-5_15

[b31] Wilke C. O. Bringing molecules back into molecular evolution. PLoS Comput Biol 8, e1002572 (2012). 22761562 10.1371/journal.pcbi.1002572 PMC3386153

[b32] Harms M. J. & Thornton J. W. Evolutionary biochemistry: revealing the historical and physical causes of protein properties. Nat Rev Genet 14, 559–571 (2013). 23864121 10.1038/nrg3540 PMC4418793

[b33] Mirny L. & Shakhnovich E. Evolutionary conservation of the folding nucleus. J Mol Biol 308, 123–129 (2001). 11327757 10.1006/jmbi.2001.4602

[b34] Mannige R. V., Brooks C. L. & Shakhnovich E. I. A Universal Trend among Proteomes Indicates an Oily Last Common Ancestor. Plos Computational Biology 8 (2012). 10.1371/journal.pcbi.1002839 PMC3531291 23300421

[b35] O'Neil K. T. & DeGrado W. F. How calmodulin binds its targets: sequence independent recognition of amphiphilic alpha-helices. Trends Biochem Sci 15, 59–64 (1990). 2186516 10.1016/0968-0004(90)90177-d

[b36] Rose G. D., Geselowitz A. R., Lesser G. J., Lee R. H. & Zehfus M. H. Hydrophobicity of amino acid residues in globular proteins. Science 229, 834–838 (1985). 4023714 10.1126/science.4023714

[b37] Benaim G. & Villalobo A. Phosphorylation of calmodulin. Functional implications. Eur J Biochem 269, 3619–3631 (2002). 12153558 10.1046/j.1432-1033.2002.03038.x

[b38] Carruthers N. J. & Stemmer P. M. Methionine oxidation in the calmodulin-binding domain of calcineurin disrupts calmodulin binding and calcineurin activation. Biochemistry 47, 3085–3095 (2008). 18275158 10.1021/bi702044x PMC4167793

[b39] Wang Q. *et al.* Protein recognition and selection through conformational and mutually induced fit. Proc Natl Acad Sci USA 110, 20545–20550 (2013). 24297894 10.1073/pnas.1312788110 PMC3870683

[b40] Levy Y., Wolynes P. G. & Onuchic J. N. Protein topology determines binding mechanism. Proc Natl Acad Sci USA 101, 511–516 (2004). 14694192 10.1073/pnas.2534828100 PMC327178

[b41] Chu X. & Wang J. Specificity and affinity quantification of flexible recognition from underlying energy landscape topography. PLoS Comput Biol 10, e1003782 (2014). 25144525 10.1371/journal.pcbi.1003782 PMC4140643

[b42] Tripathi S. *et al.* Conformational frustration in calmodulin-target recognition. J Mol Recognit 28, 74–86 (2015). 25622562 10.1002/jmr.2413 PMC4477201

[b43] Ovchinnikov S., Kamisetty H. & Baker D. Robust and accurate prediction of residue-residue interactions across protein interfaces using evolutionary information. Elife 3, e02030 (2014). 24842992 10.7554/eLife.02030 PMC4034769

[b44] Morcos F., Schafer N. P., Cheng R. R., Onuchic J. N. & Wolynes P. G. Coevolutionary information, protein folding landscapes, and the thermodynamics of natural selection. Proc Natl Acad Sci USA 111, 12408–12413 (2014). 25114242 10.1073/pnas.1413575111 PMC4151759

[b45] Krajewska W. M. & Maslowska I. Caveolins: structure and function in signal transduction. Cell Mol Biol Lett 9, 195–220 (2004). 15213803

[b46] DeWire S. M., Ahn S., Lefkowitz R. J. & Shenoy S. K. Beta-arrestins and cell signaling. Annu Rev Physiol 69, 483–510 (2007). 17305471 10.1146/annurev.physiol.69.022405.154749

[b47] Prober D. A. & Edgar B. A. Interactions between Ras1, dMyc, and dPI3K signaling in the developing Drosophila wing. Genes Dev 16, 2286–2299 (2002). 12208851 10.1101/gad.991102 PMC186666

[b48] Friedler A., Veprintsev D. B., Rutherford T., von Glos K. I. & Fersht A. R. Binding of Rad51 and other peptide sequences to a promiscuous, highly electrostatic binding site in p53. J Biol Chem 280, 8051–8059 (2005). 15611070 10.1074/jbc.M411176200

[b49] Lange O. F. *et al.* Recognition dynamics up to microseconds revealed from an RDC-derived ubiquitin ensemble in solution. Science 320, 1471–1475 (2008). 18556554 10.1126/science.1157092

[b50] Erijman A., Aizner Y. & Shifman J. M. Multispecific recognition: mechanism, evolution, and design. Biochemistry 50, 602–611 (2011). 21229991 10.1021/bi101563v

[b51] Fukuhara N. & Kawabata T. HOMCOS: a server to predict interacting protein pairs and interacting sites by homology modeling of complex structures. Nucleic Acids Res 36, W185–189 (2008). 18442990 10.1093/nar/gkn218 PMC2447736

[b52] Eswar N. *et al.* Comparative protein structure modeling using MODELLER. Curr Protoc Protein Sci Chapter 2, Unit 2 9 (2007). 10.1002/0471140864.ps0209s50 18429317

