# Supplementary material for: Lessons in Protein Design from Combined Evolution and Conformational Dynamics
Source: Sci Rep. 2015 Sep 21;5:14259. doi: 10.1038/srep14259 (PMC4585694; doi:10.1038/srep14259)
Supplement: Supplementary Information [file srep14259-s1.pdf]

Supplementary Information

Lessons in Protein Design from Combined Evolution and Conformational Dynamics

Swarnendu Tripathi [1,3], M. Neal Waxham [2], Margaret S. Cheung* [1,3], and Yin Liu* [2]

1. Department of Physics, University of Houston, Houston, TX
2. Department of Neurobiology and Anatomy, University of Texas, Health Science Center, Houston, TX
3. Center for Theoretical Biological Physics, Rice University, Houston, TX

*Corresponding authors

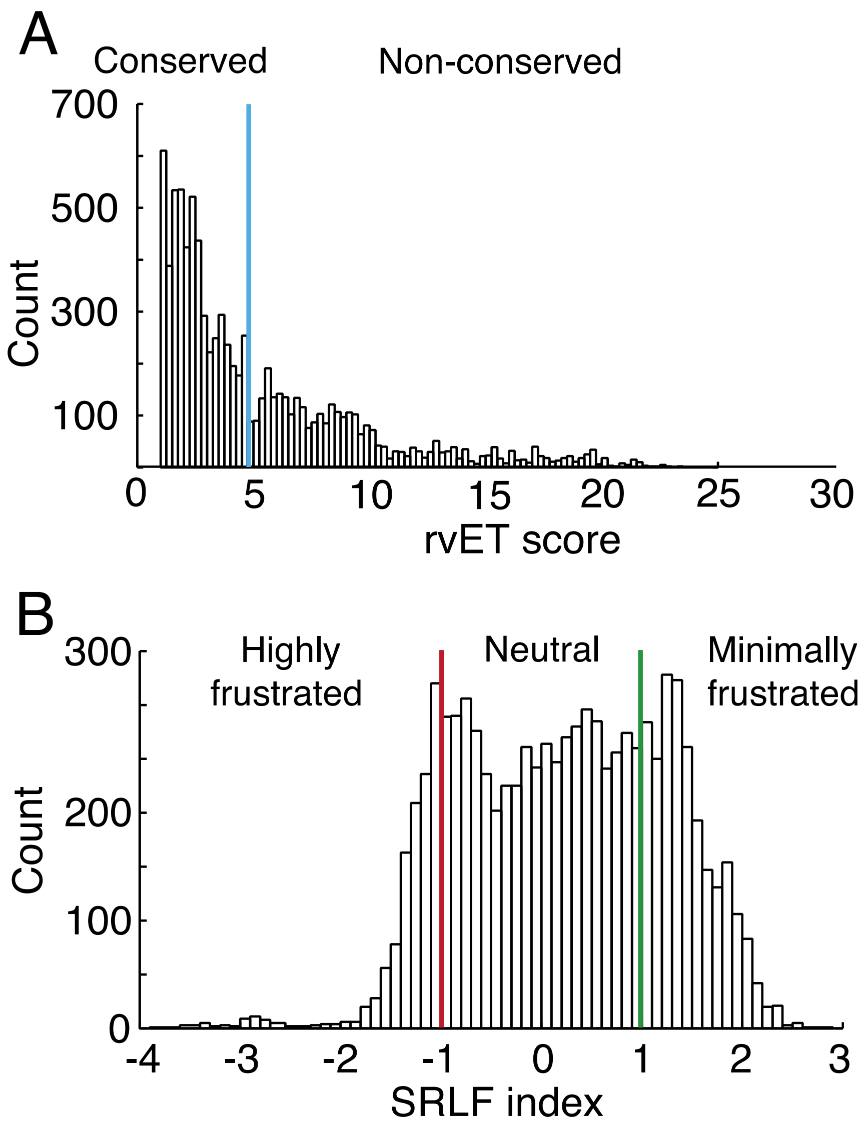

Figure S1. Distributions of evolutionary trace scores and local frustration of calmodulin (CaM) residues from 60 target bound complexes analyzed. (A) One dimensional distribution of real-value evolutionary trace (rvET) scores of all the residues of CaM. Based on the evolutionary analysis, CaM residues are divided as conserved (rvET score < 5) and non-conserved (rvET score > 5) indicated by the blue line. (B) One dimensional distribution of single residue level frustration (SRLF) indices of all the residues of CaM in the 60 complexes. Based on the local frustration analysis, CaM residues are divided as highly frustrated (SRLF index < -1, red line), minimally frustrated (SRLF index > 1, green line) and neutral (-1 < SRLF index < 1).

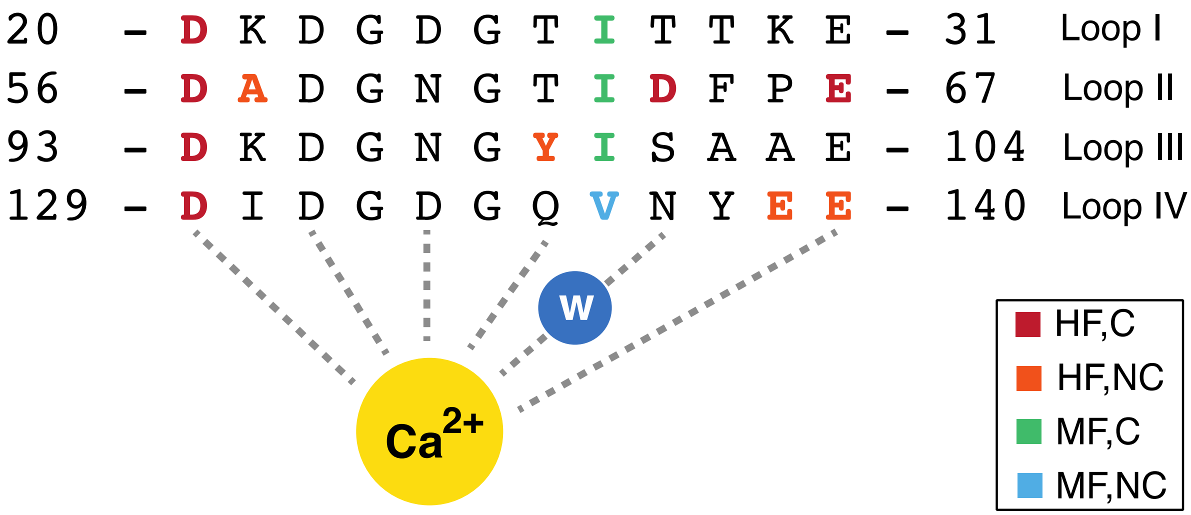

Figure S2. Sequence of the four Ca^{2+}-binding loops of CaM categorized based on the rvET score and SRLF index. The Ca^{2+} ion (shown as a yellow sphere) coordinates (indicated by grey dotted lines) with the first, third, fifth, seventh, ninth (through water molecule (w), shown as a blue sphere) and twelfth residue of the loops. The residues that fall in one of these four classes, (HF, C), (HF, NC), (MF, C) and (MF, NC) (see Table S2) are indicated by red, orange, green and blue colors, respectively and in bold letters.

```
              1         10        20        30        40        50
HUMAN         ADQLTEEQIAEFKEAFSLFDKDGDGTITTKELGTVMRSLGQNPTEAELQDMINEVDADG 59
CATTLE        ADQLTEEQIAEFKEAFSLFDKDGDGTITTKELGTVMRSLGQNPTEAELQDMINEVDADG
CHICKEN       ADQLTEEQIAEFKEAFSLFDKDGDGTITTKELGTVMRSLGQNPTEAELQDMINEVDADG
MOUSE         ADQLTEEQIAEFKEAFSLFDKDGDGTITTKELGTVMRSLGQNPTEAELQDMINEVDADG
RAT           ADQLTEEQIAEFKEAFSLFDKDGDGTITTKELGTVMRSLGQNPTEAELQDMINEVDADG
FROG          ADQLTEEQIAEFKEAFSLFDKDGDGTITTKELGTVMRSLGQNPTEAELQDMINEVDADG
FRUIT FLY     ADQLTEEQIAEFKEAFSLFDKDGDGTITTKELGTVMRSLGQNPTEAELQDMINEVDADG
BARLEY        ADQLTDEQIAEFKEAFSLFDKDGDGTITTKELGTVMRSLGQNPTEAELQDMINEVDADG
SOYBEAN       ADILSEEQIVDFKEAFGLFDKDGDGCITVEELATVIRSLDQNPTEEELQDMISEVDADG
YEAST         SSNLTEEQIAEFKEAFALFDKDNNGSISSSELATVMRSLGLSPSEAEVNDLMNEIDVDG

              60        70        80        90        100       110
HUMAN         NGTIDFPEFLTMMARKMKDTDSEEEIREAFRVFDKDGNGYISAAELRHVMTNLGEKLTDE 119
CATTLE        NGTIDFPEFLTMMARKMKDTDSEEEIREAFRVFDKDGNGYISAAELRHVMTNLGEKLTDE
CHICKEN       NGTIDFPEFLTMMARKMKDTDSEEEIREAFRVFDKDGNGYISAAELRHVMTNLGEKLTDE
MOUSE         NGTIDFPEFLTMMARKMKDTDSEEEIREAFRVFDKDGNGYISAAELRHVMTNLGEKLTDE
RAT           NGTIDFPEFLTMMARKMKDTDSEEEIREAFRVFDKDGNGYISAAELRHVMTNLGEKLTDE
FROG          NGTIDFPEFLTMMARKMKDTDSEEEIREAFRVFDKDGNGYISAAELRHVMTNLGEKLTDE
FRUIT FLY     NGTIDFPEFLTMMARKMKDTDSEEEIREAFRVFDKDGNGFISAAELRHVMTNLGEKLTDE
BARLEY        NGTIDFPEFLNLMARKMKDTDSEEELKEAFRVFDKDGNGFISAAELRHVMTNLGEKLTDE
SOYBEAN       NGTIEFDEFLSLMAKKVKDTDAEEELKEAFKVFDKDQNGYISASELRHVMINLGEKLTDE
YEAST         NHQIEFSEFLALMSRQLKSNDSEQELLEAFKVFDKNGDGLISAAELKHVLTSIGEKLTDA

              120       130       140
HUMAN         EVDEMIREADIDGDGQVNYEEFVQMMTAK- 149
CATTLE        EVDEMIREADIDGDGQVNYEEFVQMMTAK-
CHICKEN       EVDEMIREADIDGDGQVNYEEFVQMMTAK-
MOUSE         EVDEMIREADIDGDGQVNYEEFVQMMTAK-
RAT           EVDEMIREADIDGDGQVNYEEFVQMMTAK-
FROG          EVDEMIREADIDGDGQVNYEEFVQMMTAK-
FRUIT FLY     EVDEMIREADIDGDGQVNYEEFVTMMTSK-
BARLEY        EVDEMIREADVDGDGQVNYEEFVQVMMAK-
SOYBEAN       EVEQMIKEADLDGDGQVNYEEFVKMMMTVR
YEAST         EVDDMLREV-SDGSGEINIQQFAALLSK--
```

Figure S3. Multiple sequence alignment (MSA) of CaM amino acid sequences from different organisms. Our study included CaM from the following organisms (Table S1), *Homo sapiens* (human), *Bos taurus* (cattle), *Gallus gallus* (chicken), *Mus musculus* (mouse), *Rattus norvegicus* (rat), *Xenopus laevis* (frog), *Drosophila melanogaster* (fruit fly), *Hordeum vulgare* (barley), *Glycine max* (soybean) and *Saccharomyces cerevisiae* (baker's yeast). The organisms shown in bold letters have identical CaM sequences. The positively charged residues, negatively charged residues, hydrophobic residues, and other residues are shown in magenta, blue, red and green color, respectively. MSA was performed using the ClustalW server (http://www.ebi.ac.uk/Tools/msa/clustalw2/).

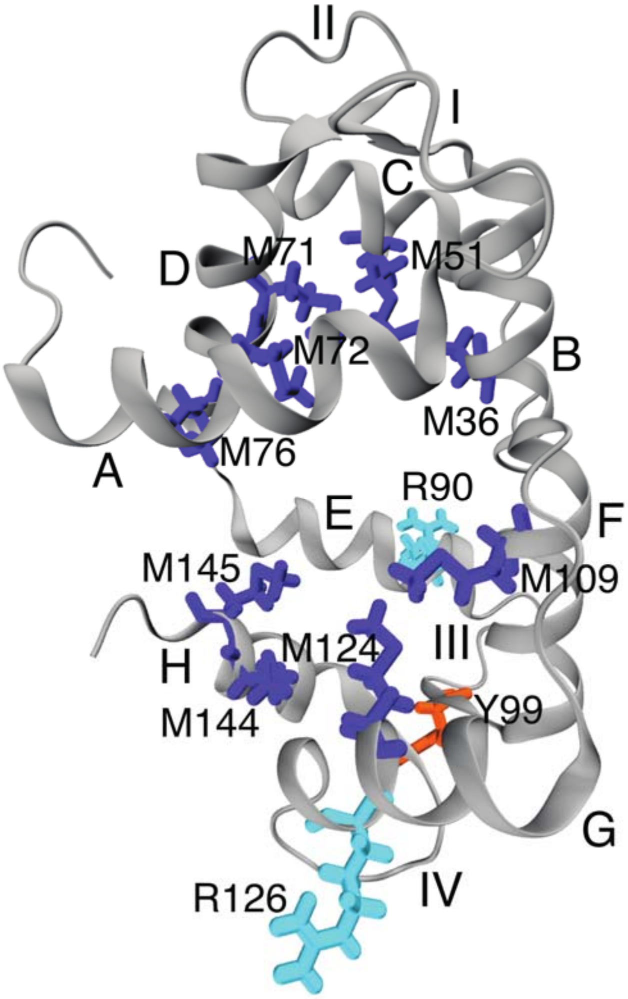

Figure S4. Structure of CaM representing the position of specific amino-acid residues analyzed in Fig. S5-S8. Tyr99 (in orange), Arg90, Arg126 (in cyan) and nine methionine residues (Met at 36, 51, 71, 72, 76, 109, 124, 144 and 145 in blue) are shown in “licorice” representation. The structure of CaM is from the CaM-CaMKI complex (PDB ID: 2L7L) and CaMKI is not shown in the above structure.

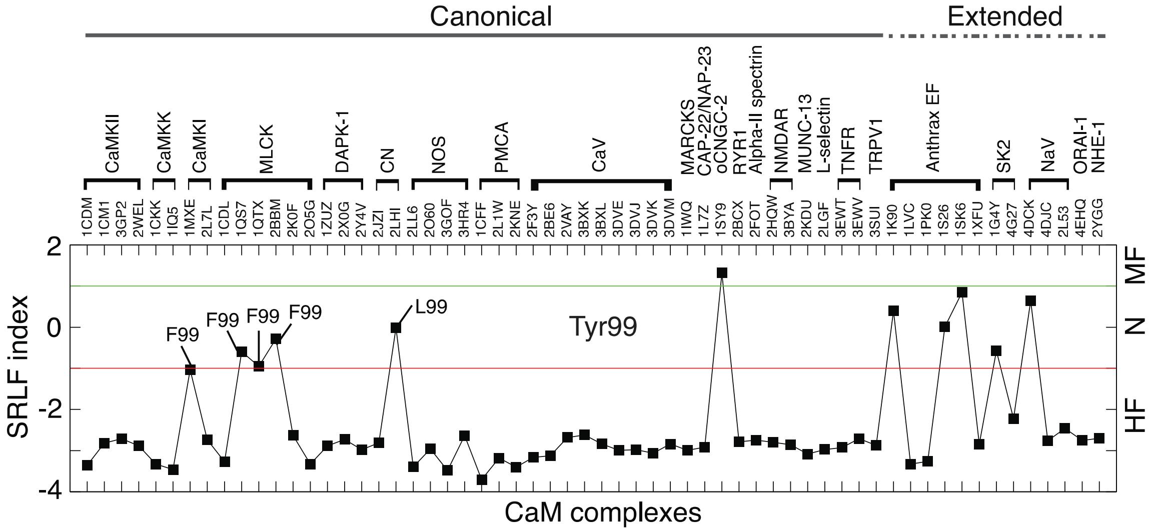

Figure S5. Single residue level frustration (SRLF) of Tyr at position 99 (of CaM) in 60 CaM complexes. The PDB codes of each complex, along with the names of the corresponding target proteins are indicated at the top panel. The binding mode of CaM is categorized as canonical or extended. Notice that the lines connecting the individual points are for visualization purposes only and are not meant to imply a connection between the individual complexes. The amino acid variation at position 99 of CaM, includes Y99/F99 in fruit fly (1MXE and 2BBM) or barley (1QS7 and 1QTX), and Y99/L99 in baker's yeast (2LHI) are indicated inside the plot. Horizontal green and red lines separate the regions into minimally frustrated (MF), neutral (N) and highly frustrated (HF). When Tyr99 is replaced with one of these hydrophobic residues the frustration index goes from high to neutral.

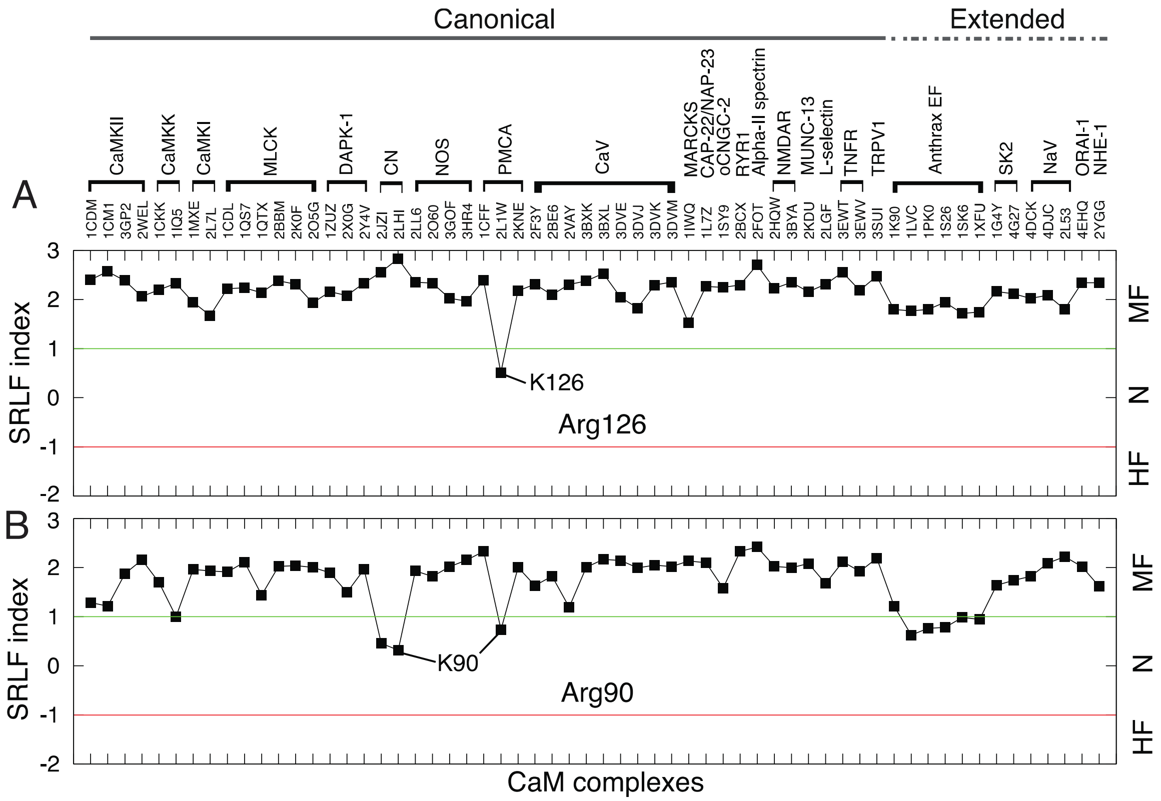

Figure S6. Single residue level frustration (SRLF) of Arg at positions 90 and 126 (of CaM) in the 60 CaM complexes. (A) SRLF of Arg126. (B) SRLF of Arg90. Notice that the lines connecting the individual points are for visual guidance only. The changes of R126/K126 in soybean (2L1W) in (A) and R90/K90 in baker's yeast (2LHI) or soybean (2L1W) in (B) produce an increase in frustration from minimal to neutral.

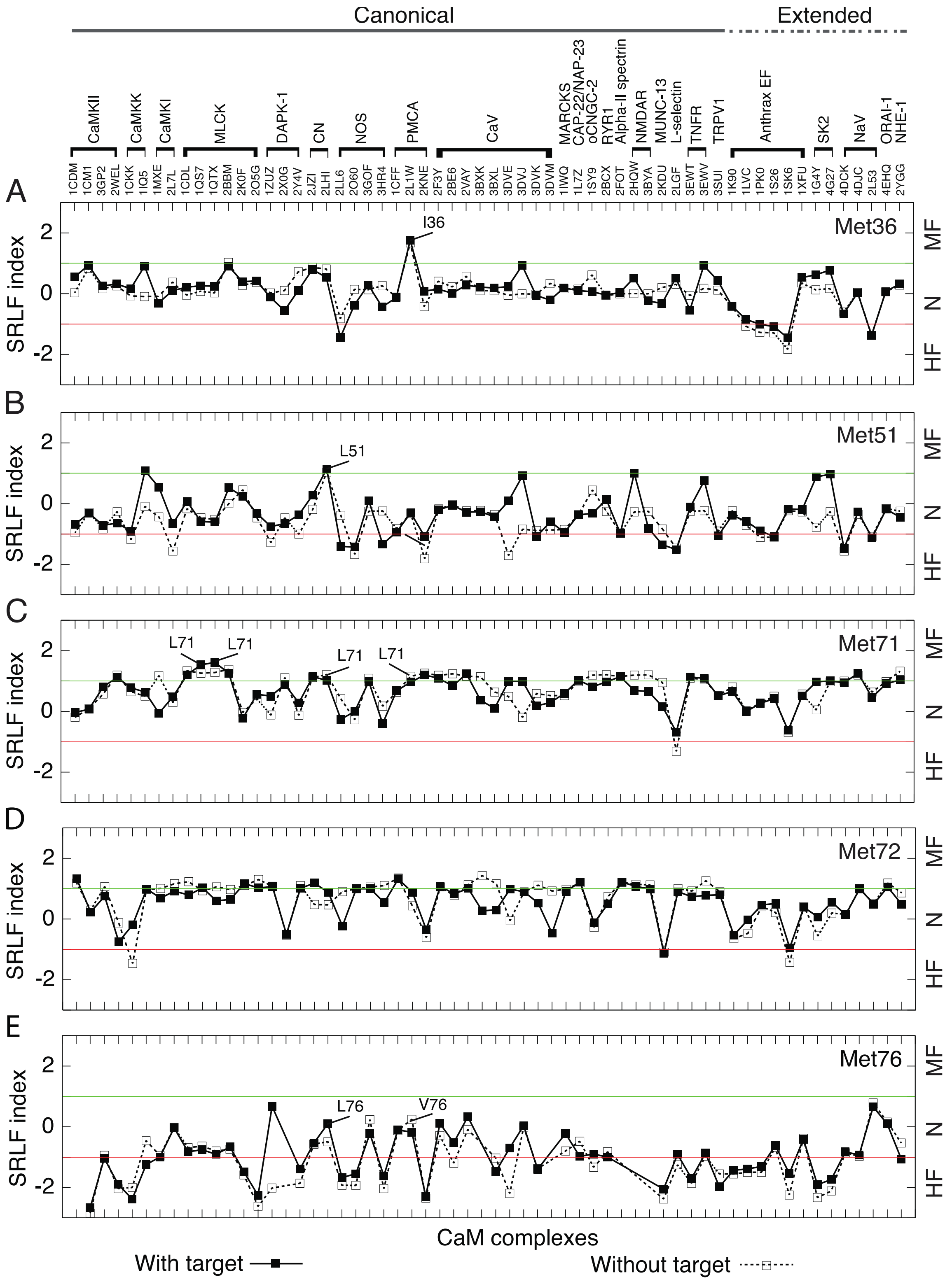

Figure S7. Single residue level frustration (SRLF) of Met residues in 60 CaM complexes. SRLF was calculated both in the presence and absence (by removing the target from the

CaM complex) of the target for each CaM complex (see *Methods* in the main text). SRLF of Met36 (A), Met51 (B), Met71 (C), Met72 (D), and Met76 (E) are shown in the respective plots. Notice that the lines connecting the individual points are for visual guidance only. The changes in Met residues of CaM, including M36/I36 (2L1W from soybean), M51/L51 (2LHI from baker's yeast), M71/L71 (1QS7 and 1QTX from barley, and 2LHI from baker's yeast and 2L1W from soybean), M76/L76 (2LHI from baker's yeast) and M76/V76 (2L1W from soybean) all lead to decreases in frustration levels (towards MF). Overall, there is little difference in the frustration level of these Met residues when calculations were made in the presence (filled symbols) or absence (unfilled symbols) of target except for Met51. In nine complexes, the frustration of Met51 decreases (SRLF index is increased by more than one standard deviation of the frustration indices) upon target binding.

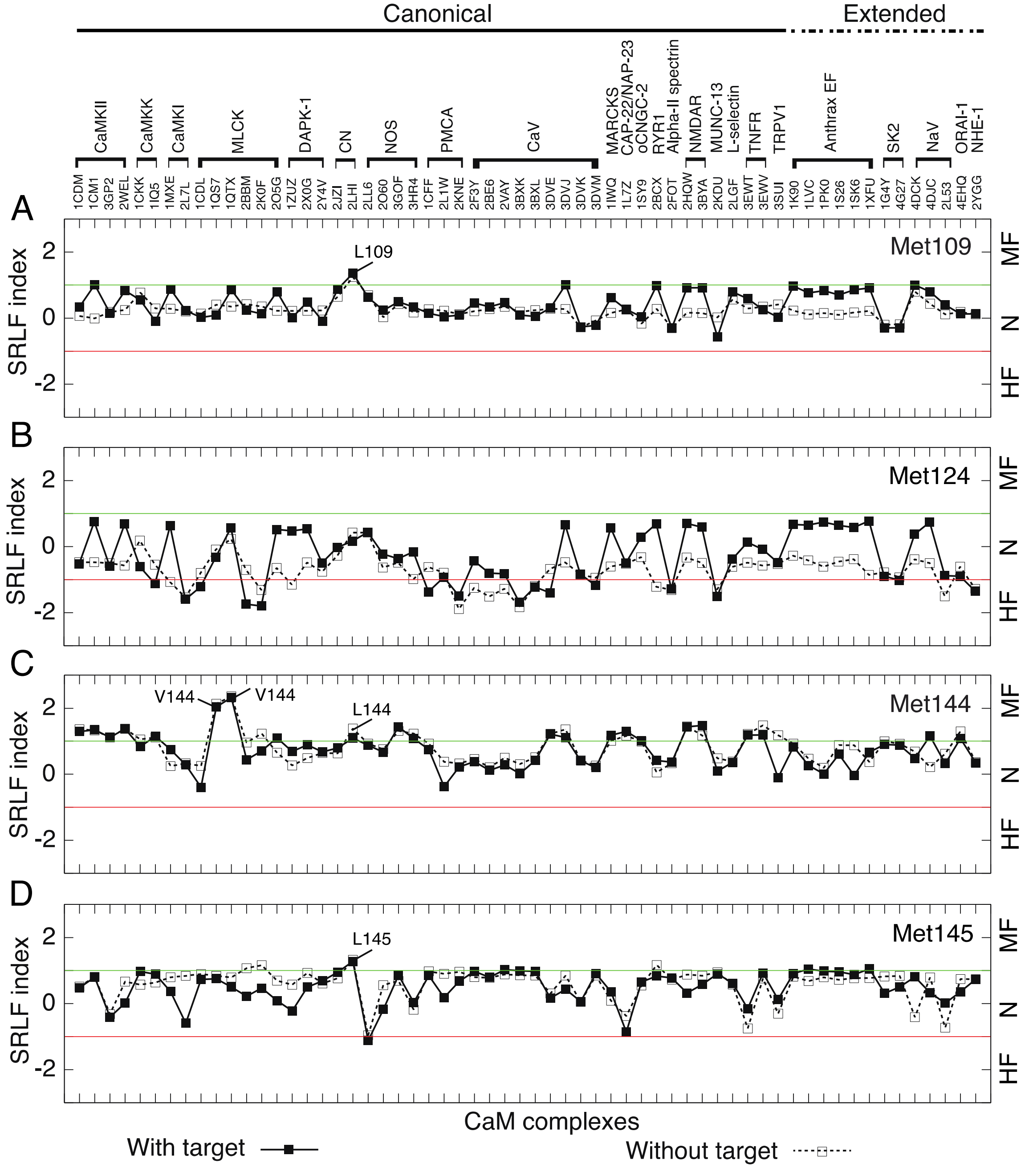

Figure S8. Single residue level frustration (SRLF) of Met residues in 60 CaM complexes. SRLF was calculated both in the presence and absence (by removing the target from the CaM complex) of the target for each CaM complex (see *Methods* in the main text). SRLF of Met109 (A), Met124 (B), Met144 (C), and Met145 (D) are shown in the respective plots. Notice that the lines connecting the individual points are for visual guidance only. The

changes in Met residues of CaM, M109/L109 (2LHI from baker's yeast), M144/V144 (1QS7 and 1QTX from barley, and 2LHI from baker's yeast), and M145/L145 (2LHI from baker's yeast) all lead to decreases in frustration index (towards MF). Overall, there is little difference in the frustration level of these Met residues when calculations were made in the presence (filled symbols) or absence (unfilled symbols) of target, except for Met124. The frustration level of Met124 significantly decreases (SRLF index is increased by more than one standard deviation of the frustration indices) in 16 complexes and increases in another 4 complexes in the presence of target.

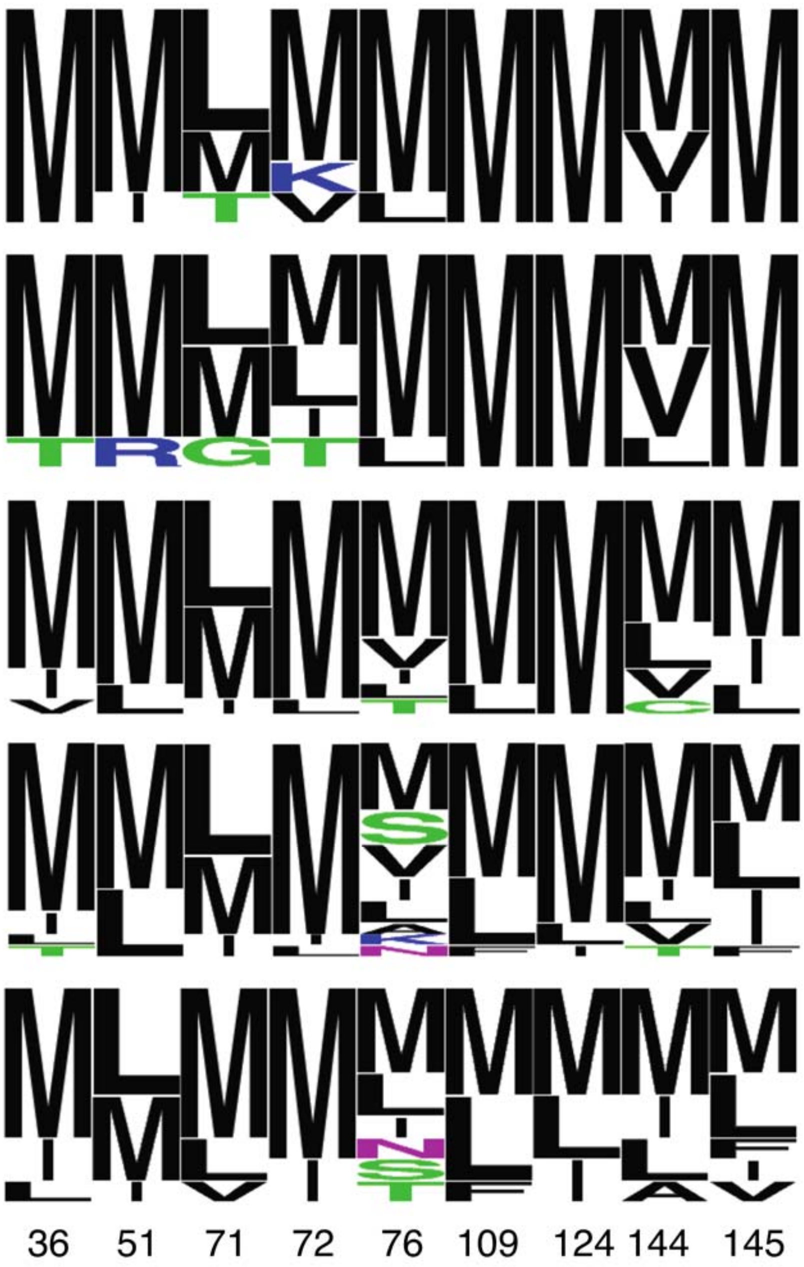

Figure S9. Variation of Met residues at the positions 36, 51, 71, 72, 76, 109, 124, 144, and 145 of CaM based on homologous CaM sequences. Homologous CaM sequences were obtained from BLAST search. All hypothetical, predicted or non-CaM protein sequences were removed from BLAST results before the sequences were subjected to multiple sequence alignment analysis. The distance between each CaM homologous sequence and human CaM was then calculated, where the distance indicates the number of amino acid substitutions as a proportion of the length of the sequence alignment (excluding gaps). The

resulting distance values range between 0 and 0.5. The homologous sequences of CaM were then grouped into five distance bins from the least (top, with distance < 0.1) to the most (bottom, with 0.4 < distance < 0.5) divergent, according to their distances from human CaM. The sequence profiles of the nine Met residues in each distance bin were represented by the sequence logos generated from the WebLogo tool (http://weblogo.berkeley.edu/logo.cgi). It is noted Met124 is the most conserved Met residue in CaM and only two variants, Leu and Ile exist at this position in lower eukaryotes, whose CaM sequences include more than 40% amino acid substitutions compared to human CaM.

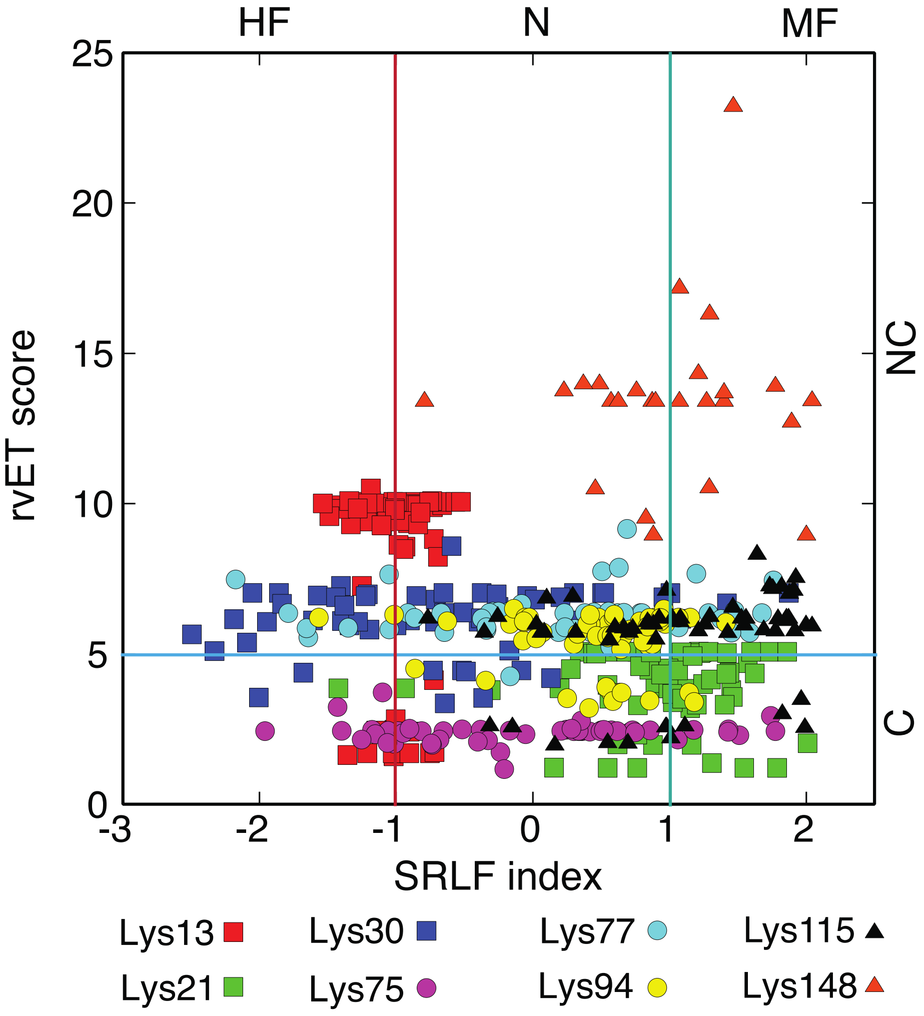

Figure S10. Evolutionary trace and local frustration of Lys residues of CaM. rvET score vs. SRLF index plot of eight Lys residues of CaM at positions 13, 21, 30, 75, 77, 94, 115, and 148 are shown.

Table S1. List of 60 CaM-target protein complexes.

PDB	Target protein	CaM organism	Target organism	Number of Ca^{2+}	Method
Canonical Binding mode					
1CDM	CaM-dependent protein kinase II	Bos taurus	Bos taurus	4	X-ray
1CM1	CaM-dependent protein kinase II alpha	Bos taurus	Bos taurus	4	X-ray
3GP2	CaM-kinase II delta	Gallus gallus	Homo sapiens	4	X-ray
2WEL	CaM-dependent protein kinase II delta	Homo sapiens	Homo sapiens	4	X-ray
1CKK	Ca2+/CaM-dependent protein kinase kinase	Xenopus laevis	Rattus norvegicus	4	NMR
1IQ5	Ca2+/CaM-dependent protein kinase kinase	Xenopus laevis	Caenorhabditis elegans	4	X-ray
1MXE	CaM-dependent protein kinase I	Drosophila melanogaster	Rattus norvegicus	4	X-ray
2L7L	CaM-kinase I alpha	Homo sapiens	Rattus norvegicus	4	NMR
1CDL	MLCK	Homo sapiens	Gallus gallus	4	X-ray
1QS7	MLCK	Hordeum vulgare	Gallus gallus	4	X-ray
1QTX	MLCK	Hordeum vulgare	Gallus gallus	4	X-ray
2BBM	MLCK	Drosophila melanogaster	Oryctolagus cuniculus	4	NMR
2K0F	MLCK	Homo sapiens	Homo sapiens	4	NMR
2O5G	MLCK	Gallus gallus	Gallus gallus	4	NMR
1ZUZ	DAPK-1	Homo sapiens	Homo sapiens	4	X-ray
2Y4V	DAPK-1	Homo sapiens	Homo sapiens	4	X-ray
2JZI	Calcineurin A alpha	Homo sapiens	Homo sapiens	4	NMR
2LHI	Calcineurin A1	Saccharomyces cerevisiae	Saccharomyces cerevisiae	3	NMR
2LL6	NOS	Homo sapiens	Homo sapiens	0	NMR
2O60	NOS	Gallus gallus	Mus musculus	4	X-ray
3GOF	NOS	Gallus gallus	Mus musculus	4	X-ray
3HR4	NOS	Homo sapiens	Homo sapiens	4	X-ray

1CFF	PMCA	Xenopus laevis	Homo sapiens	4	NMR
2L1W	Vacuolar Ca2+-ATPase	Glycine max	Glycine max	4	NMR
2KNE	PMCA4	Homo sapiens	Homo sapiens	4	NMR
2F3Y	CaV1.2 (IQ-motif)	Homo sapiens	Cavia porcellus	4	X-ray
2BE6	CaV1.2 (IQ-domain)	Homo sapiens	Homo sapiens	4	X-ray
2VAY	CaV1.1 (IQ-domain)	Homo sapiens	Homo sapiens	4	X-ray
3BXK	CaV2.1	Rattus norvegicus	Homo sapiens	4	X-ray
3BXL	CaV2.3	Rattus norvegicus	Homo sapiens	4	X-ray
3DVE	CaV2.2	Homo sapiens	Oryctolagus cuniculus	4	X-ray
3DVJ	CaV2.2	Homo sapiens	Oryctolagus cuniculus	4	X-ray
3DVK	CaV2.3	Homo sapiens	Rattus norvegicus	4	X-ray
3DVM	CaV2.1	Homo sapiens	Oryctolagus cuniculus	4	X-ray
1IWQ	Myristoylated alanine-rich C-kinase substrate	Homo sapiens	Mus musculus	4	X-ray
1L7Z	CAP-22/NAP-23	Homo sapiens	Homo sapiens	4	X-ray
1SY9	Olfactory CNG channel 2	Xenopus laevis	Bos taurus	4	NMR
2BCX	Ryanodine receptor 1	Gallus gallus	Oryctolagus cuniculus	4	X-ray
2FOT	Alpha-II spectrin	Bos taurus	Homo sapiens	4	X-ray
2HQW	NMDA receptor	Rattus norvegicus	Homo sapiens	4	X-ray
3BYA	NMDA receptor	Homo sapiens	Homo sapiens	4	X-ray
2KDU	MUNC-13	Xenopus laevis	Rattus norvegicus	4	NMR
2LGF	l-selectin	Homo sapiens	Homo sapiens	4	NMR
3EWT	Tumour necrosis factor receptor 6	Homo sapiens	Homo sapiens	4	X-ray
3EWV	Tumour necrosis factor 16 receptor	Homo sapiens	Homo sapiens	4	X-ray
3SUI	TRP channel (TRPV1)	Homo sapiens	Rattus norvegicus	4	X-ray

Extended Binding Mode					
1K90	Anthrax EF	Homo sapiens	Bacillus anthracis	2	X-ray
1LVC	Anthrax EF	Homo sapiens	Bacillus anthracis	2	X-ray
1PK0	Anthrax EF	Homo sapiens	Bacillus anthracis	2	X-ray
1S26	Anthrax EF	Homo sapiens	Bacillus anthracis	2	X-ray
1SK6	Anthrax EF	Homo sapiens	Bacillus anthracis	2	X-ray
1XFU	Anthrax EF	Homo sapiens	Bacillus anthracis	3	X-ray
1G4Y	SK2	Rattus norvegicus	Rattus norvegicus	2	X-ray
4G27	SK2	Rattus norvegicus	Rattus norvegicus	2	X-ray
4DCK	NaV1.5 (+FGF13)	Homo sapiens	Homo sapiens	0	X-ray
4DJC	NaV1.5	Homo sapiens	Homo sapiens	4	X-ray
2L53	NaV1.5 (IQ-motif)	Homo sapiens	Homo sapiens	0	NMR
4EHQ	ORAI-1	Rattus norvegicus	Homo sapiens	4	X-ray
2YGG	Na+/H+-exchanger NHE-1	Rattus norvegicus	Homo sapiens	4	X-ray

Table S2. Classification of Calmodulin residues. Each residue of CaM was categorized into one of six classes based on both its degree of conservation (conserved (C) or non-conserved (NC)) and its frustration level (highly frustrated (HF), neutral (N) or minimally frustrated (MF). in different CaM-target complexes. A residue had to be found in at least 60% of the complexes to be designated as belonging to one of the classes.

Classes	CaM Residues
HF, C<br>(7 residues)	D20, D56, D64, E67, D93, E123, D129
N, C<br>(47 residues)	A15, D22, D24, G25, T28, T29, G33, M36, R37, S38, G40, Q41, N42, E45, E47, D50, M51, E54, D58, N60, G61, M72, A73, K75, D80, E82, E83, E84, A88, F92, D95, N97, S101, E104, R106, H107, M109, G113, E114, E120, M124, E127, A128, D131, G132, D133, G134
MF, C<br>(20 residues)	F16, L18, F19, I27, L32, T34, V35, L39, L48, I52, V55, I63, F68, L69, V91, I100, L105, L112, L116, V121
HF, NC<br>(5 residues)	A57, Y99, E119, E139, E140
N, NC<br>(33 residues)	E6, E7, Q8, A10, E11, S17, G23, T26, T44, A46, Q49, N53, G59, T62, T70, M71, R74, T79, S81, K94, A103, T110, T117, D118, D122, I130, Q135, N137, Y138, Q143, M144, M145, T146
MF, NC<br>(12 residues)	L4, T5, I9, F12, I85, R90, V108, I125, R126, V136, F141, V142
